# Effects of Zoledronic Acid on Physiologic Bone Remodeling of Condylar Part of TMJ: A Radiologic and Histomorphometric Examination in Rabbits

**DOI:** 10.1155/2014/649026

**Published:** 2014-01-21

**Authors:** Ufuk Tatli, Yakup Üstün, Mehmet Kürkçü, Mehmet Emre Benlidayı

**Affiliations:** ^1^Department of Oral and Maxillofacial Surgery, Faculty of Dentistry, Çukurova University, Saricam-Balcali, 01330 Adana, Turkey; ^2^Private Practice in Oral and Maxillofacial Surgery, 01120 Adana, Turkey

## Abstract

*Objective*. The purpose of the present study is to evaluate the effects of systemically administered zoledronic acid (ZA) on the physiological bone remodeling and the microarchitectural parameters of the condylar part of TMJ in a rabbit model. *Study Design*. Thirty skeletally mature male New Zealand white rabbits were randomly divided into two groups. The experimental group was administered an intravenous, single dose of 0.1 mg/kg ZA diluted with 15 mL of saline in a 15-minute perfusion with an infusion pump. The control group was administered only saline infusion for 15 minutes. All rabbits were sacrificed on the 21st postoperative day. Radiodensitometric and histomorphometric examinations were performed on the harvested mandibular condyles. The data were analyzed statistically. *Results*. Radiodensitometric findings showed that ZA treatment resulted in a significant increase in the mineralization of mandibular condyle. This result was supported by the histomorphometric findings. *Conclusion*. The present study has revealed that a temporary delay in the physiological bone remodeling using single dose of ZA increases bone mineral content and makes the microarchitecture of the mandibular condyle more compact. These effects may be regarded as base data and considered in numerous clinical situations including TMJ.

## 1. Introduction

The bony components of the temporomandibular joint (TMJ) are the articular fossa and articular eminence of temporal bone and mandibular condyle. Under normal physiologic conditions, a balance exists in synovial joints between tissue breakdown and repair. When the balance is disturbed by a mechanical, biomechanical, or inflammatory insult, the internal cartilaginous remodeling system may fail, resulting in accelerated tissue breakdown and articular bone resorption [1]. In the resorption phase, catabolic activities preponderate over anabolic responses resulting in radiographically visible degenerative changes such as flattening, sclerosis, or osteophyte in the articular bony areas [2]. As such, the amount of bone tissue could theoretically be bolstered by increasing anabolism or decreasing catabolism or both. Since the condylar bone is a load-bearing part of TMJ, remodeling process of the condyle is important in preventing microdamage accumulation as a consequence of repetitive loading during jaw moment and clenching [3, 4]. In recent years there has been increased interest in the effects of antiresorptive therapies on trabecular architecture. Suppression of bone turnover using antiresorptive agents such as bisphosphonates (BPs) prevents bone loss but may also increase tissue mineralization [5].

BPs are a group of synthetic analogs of inorganic pyrophosphate, an endogenous regulator of bone mineralization [6]. BPs are well-recognized inhibitors of osteoclastic activity and have widely been used in the clinical treatment of various systemic metabolic bone diseases. Current indications include Paget's disease [7], hypercalcemia of malignancy [8], postmenopausal osteoporosis [9], fibrous dysplasia [10], osteogenesis imperfect [11], osteoarthritis [12], and rheumatoid arthritis [13]. Zoledronic acid (ZA), a new generation of intravenous BPs, has exhibited the greatest affinity for bone mineral with the longest retention [6]. Nowadays a novel effect of BPs on bone healing has been defined. Researchers showed that single dose of ZA in rabbits improved bone healing during distraction osteogenesis [14], osseointegration period of dental implants [15], and fracture healing [16] in maxillofacial area.

Published information is lacking on the physiologic trabecular bone remodeling (TBR) in the mandibular condyle, as well as the effects of BP therapy on this condylar TBR [3]. Physiologic bone remodeling and architecture and density of the condylar subchondral bone are continuously constructed to withstand the mechanical forces and to accommodate the stress on the fibrocartilage [17]. Thus, understanding of changes occurring in physiologic bone remodeling of mandibular condyle after BP administration is crucial in the future development of treatment modalities of degenerative TMJ diseases causing condylar bone resorption.

With this background, the purpose of the present study is to evaluate the effects of systemically administered ZA on the physiological bone remodeling and the microarchitectural parameters of the condylar part of TMJ in a rabbit model using radiodensitometric and histomorphometric methods.

## 2. Materials and Methods

The ethical review committee of Çukurova University Medical Scientific Research Center approved the study. The experimental procedures and care of animals were in accordance with the European Convention for the Protection of Vertebrate Animals used for Experimental Scientific Purposes. A total of 30 skeletally mature, male New Zealand, white rabbits, weighing from 2.8 to 3.4 kg (mean 3.15 ± 0.25), were included in the study. The rabbits were randomly divided into two groups. The experimental group received a single intravenous infusion of 0.1 mg/kg ZA (Zometa; Novartis, Istanbul, Turkey) diluted with 15 mL of saline in a 15-minute perfusion with an infusion pump. The control group received a saline infusion only for 15 minutes. All the rabbits received the drug under general anesthesia, obtained by intramuscular injection of 35 mg/kg ketamine (Ketalar; Pfizer, Istanbul, Turkey) and 3 mg/kg xylazine (Rompun; Bayer, Istanbul, Turkey). Then, the rabbits were kept in separate cages. The food and water intake and weight of the rabbits were recorded daily.

**Figure 1:**
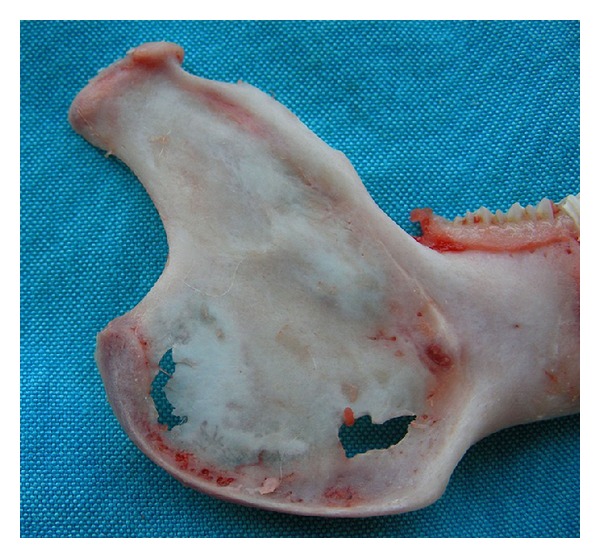
Subperiosteally dissected hemimandible of the rabbit including condyle.

No surgical intervention was performed in the rabbits to see the isolated BP effect on the physiological bone remodeling of the mandibular condyle without cofactors (steroids, TMJ surgery, etc.). Twenty-one days after the ZA infusion, all the rabbits were killed by an intravenous injection of 100 mg/kg sodium pentobarbitone (Pental; IE Ulagay, Istanbul, Turkey), and the mandibles were dissected subperiosteally. The mandibles were split at the midline. Thus, two hemimandibles including condyles were obtained from each rabbit (Figure 1). The condyles were resected from the subcondylar region and the samples were wrapped in saline-soaked gauze and stored at −20°C until the examinations.

### 2.1. Radiographic Examination

**Figure 2:**
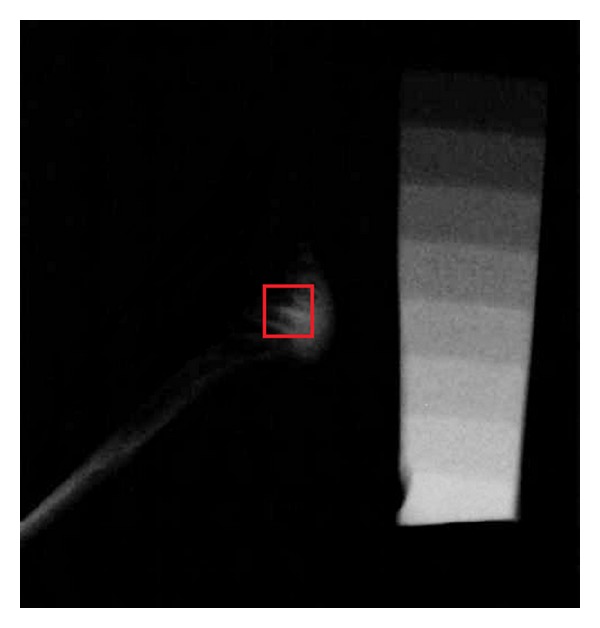
Radiographic image of rabbit condyle and aluminum step wedge from control group.

**Figure 3:**
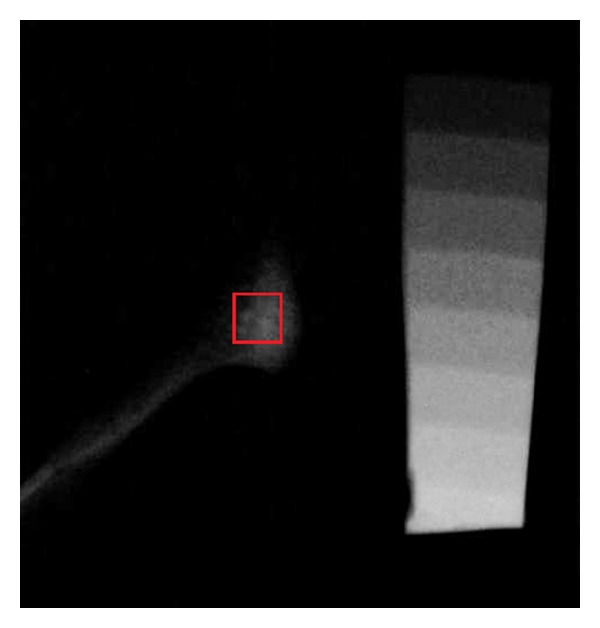
Radiographic image of rabbit condyle and aluminum step wedge from ZA-treated group.

Digital radiographs of all the condyles were taken from the lateral aspect, with an aluminum step wedge attached to the sensor of the digital radiography device (RVG, Trophy Radiologie, Vincennes, France). The aluminum step wedge consisted of 10 steps, with a thickness of 1 to 10 mm. The same aluminum step wedge was used for all radiographs. The X-ray unit (Philips Densomat, Eindhoven, The Netherlands) was set at 65 kVp, 300 mA, and 0.16 ms. The X-ray cone was directed perpendicularly to the sensor from a distance of 20 cm. The digital images were converted to “tiff” format using imaging software (Adobe Photoshop CS2; Adobe Systems, San Jose, CA, USA) and a standardized measurement area (2 × 2 mm) was outlined in the middle of the condylar bone (Figures 2 and 3). The bone density was measured using image analyzing software (ImageJ, version 1.33u; Wayne Rasband, National Institutes of Health, Bethesda, MD, USA). The gray level of each step of the aluminum step wedge was measured and used for calibration of the software. The aluminum-equivalent bone density of the condylar bone was measured. The results were expressed as millimeters of aluminum.

### 2.2. Histomorphometric Examination

**Figure 4:**
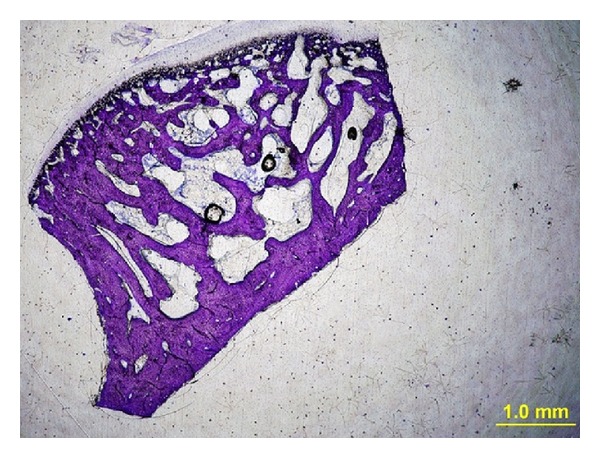
A 50 *μ*m thick histologic section prepared for histomorphometric analysis from control group (toluidine blue stain, original magnification ×2).

**Figure 5:**
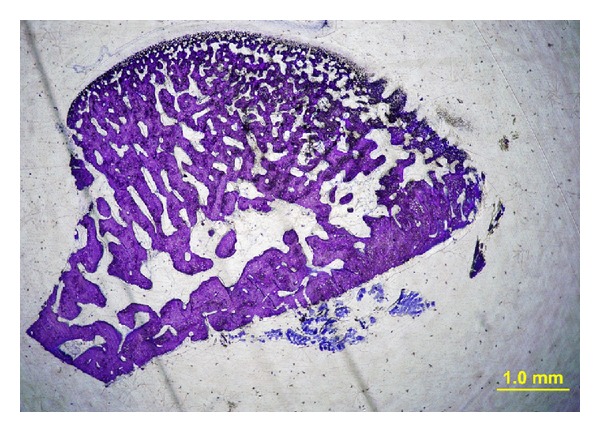
A 50 *μ*m thick histologic section prepared for histomorphometric analysis from ZA-treated group (toluidine blue stain, original magnification ×2).

Undecalcified sections of 30 intact samples from each group were prepared. The histomorphometric examination was performed as described by our previous study [16]. The specimens were fixed in 10% buffered formalin, dehydrated in increasing concentrations of ethanol of 70% to 99% for 10 days, and embedded in methylmethacrylate (Technovit 7200VLC; Heraeus Kulzer GmbH, Wehrheim, Germany). The 50 *μ*m thick sagittal sections were prepared using an electric diamond saw and grinding system (Exakt; Exakt Vertriebs, Norderstedt, Germany) and stained with toluidine blue. Digital images of the sections were obtained using a digital camera (Camedia C4040; Olympus, Tokyo, Japan) attached to an Olympus BX50 microscope (Olympus) at a magnification rate of 2x (Figures 4 and 5). The images were transferred to a personal computer, and a standardized measurement area (2 × 2 mm) was outlined in the middle of the condylar bone. Bone volume, trabecular width, trabecular thickness, trabecular separation, and node/terminus ratio measurements were made using histomorphometry software (TAS, version 1.2.9; Steve Paxton, University of Leeds, Leeds, West Yorkshire, UK). The nomenclature and calculations for bone histomorphometry were applied in accordance with the report from the American Society for Bone and Mineral Research [18].

### 2.3. Statistical Analysis

Statistical analysis was performed with SPSS software, version 11.5 (SPSS, Chicago, IL). The data from the radiographic and histomorphometric evaluations were statistically analyzed using the unpaired *t*-test (Student's *t*-test). *P* < 0.05 was considered significant.

## 3. Results

The rabbits developed no complication during the study period. All the rabbits were considered for evaluation.

### 3.1. Radiographic Analysis

The mean aluminum thickness equivalent of the gray pixel value at the condylar area was 5.535 ± 2.754 mm of aluminum for the control group and 9.676 ± 3.475 mm of aluminum for the ZA-treated group (Table 1). The bone density was 1.74 times increased in the ZA-treated group and the difference between the two groups was statistically significant (*P* = 0.001).

### 3.2. Histomorphometric Analysis

The histomorphometric data were listed in Table 2. The differences in bone volume, trabecular width, trabecular thickness, trabecular separation, and node/terminus ratio between the two groups were statistically significant (*P* = 0.001, *P* = 0.001, *P* = 0.009, *P* < 0.001, and *P* = 0.024, resp.). In terms of bone microarchitecture, bone volume was 1.3 times, trabecular width was 1.42 times, trabecular thickness was 1.39 times, and node/terminus ratio was 1.58 times increased; on the contrary, trabecular separation was 0.47 times decreased in the ZA-treated condyles.

## 4. Discussion

Bone resorption occurs on the condylar part of TMJ in pathological conditions such as excessive trauma and inflammation [2]. The main goal of the treatment of degenerative and osteoarthritic changes of TMJ is to resolve the inflammatory resorptive activity at the articular region. BPs have become the primary therapy for treating diseases of unbalanced bone resorption [6]. In vivo bone turnover is determined by a delicate balance between osteoclastic bone resorption and osteoblastic bone formation. von Knoch et al. [19] suggest that BPs impact both sides of this balance: inhibit osteoclastic activity and have an anabolic effect on osteoblasts. Suppression of bone remodeling was demonstrated in dogs on BP therapy, with greater suppression at sites with higher levels of physiologic remodeling [20]. So, we hypothesized that high local bone turnover particular to the condylar bone may be shifted towards a positive balance by an adjunct BP therapy in the condylar part of the TMJ.

The underlying molecular mechanism in nitrogen-containing BPs, such as alendronate and risedronate, is the inhibition of enzymes in the mevalonate pathway of cholesterol synthesis that are essential for osteoclast activity and survival [21]. Consequently, BPs inactivate osteoclasts, which then undergo apoptosis, resulting in reduced bone resorption, lower bone turnover, and a positive bone balance [21]. Another pharmacologic action of BPs is the proliferation and maturation of osteoblasts [19]. Naidu et al. [22] reported that lower concentrations of BPs had a beneficial effect on osteoblast viability and function. Thus, reduced bone turnover allows more time for mineralization of existing bone, increasing the bone density [6]. BP treatment leads to the retention of trabeculae that act as a scaffold for more bone to be deposited on [23]. In the light of radiodensitometric analysis, the present study indicated a significantly greater amount of mineralized bone (1.74 times greater) in the ZA-treated group. In the literature, it was reported that the experimental TMJ arthritis resulted in low degree of mineralization compared to healthy condyles and was associated with morphological changes [24]. According to this background with the result of the present study, adjunct antiresorptive effects of ZA may theoretically support the treatment of TMJ arthritis by improving bone mineralization. Further studies involving samples with experimentally induced degenerative TMJ disorders are necessary in order to make more clear comments.

Microarchitecture is an important element of bone quality. Thus, the assessment of bone microarchitecture is crucial in evaluating the effects of adjunct antiresorptive drug therapies. Several methods are available to assess the bone architecture, particularly at the trabecular level, including histomorphometry, quantitative computed tomography, high-resolution computed tomography, volumetric quantitative computed tomography, and high-resolution magnetic resonance imaging [25, 26]. In the present study, the quantitative assessment of the condylar bone was performed using the histomorphometric method. Histomorphometric examination allows the measurement of the trabecular profiles and the count of their connections on two-dimensional sections. Recent observations seem to confirm that microstructural alterations are important determinants of bone strength, independently of bone density [27]. Trabecular separation has been defined as the distance between the edges of the trabeculae [18]. The ratio between the nodes and termini in a section is an index of the spatial connectivity in the trabecular network [27]. In the light of histomorphometric analysis, the present study showed that the administration of single dose ZA made the microarchitecture of the mandibular condyle more compact in rabbits. In an experimental study in dogs, Helm et al. [3] demonstrated that a total of 4 infusions of ZA administered monthly resulted in reduction in trabecular bone remodeling of mandibular condyle. However, the authors of the aforementioned study did not report significant difference in dogs between ZA-treated and control groups in terms of microarchitectural parameters. This might be due to different animal models.

The increase in the mechanical fixation of metallic biomaterials (metallic joint prosthesis, plate, and screws) in bone is considered an important factor in terms of treatment success. Tengvall et al. [28] demonstrated that surface treatment with BPs improved the mechanical fixation of stainless-steel screws. Consequently, BPs could also be used to improve the fixation of prosthetic joint replacement components in the surrounding bone. Further studies involving the samples with prosthetic joint replacements are necessary in order to make more clear comments about this phenomenon.

The levels of physiologic bone remodeling differ among types of bone, skeletal sites, and regions within skeletal sites, as well as with age [3]. The jawbones might be more affected than other parts of the skeletal system because of the increased bone remodeling that occurs around teeth in the alveolar region [29]. Mandibular condyle is an important growth center and also functions as an articular structure that resists compressive forces [3]. This might result in an excessive amount of BPs deposited in these mentioned regions. Therefore, the positive and negative effects of BPs in such regions must be well recognized in maxillofacial practice. The present study is the first investigation in which the effects of single dose of ZA on physiological bone remodeling of the condylar part of TMJ were evaluated.

BPs have a well-documented profile of possible side effects. An initial influenza-like illness has been documented with the first infusion of BPs. Renal failure has been noted in patients with cancer after repetitive high-dose infusions [30]. Recently, an association between BPs and osteonecrosis of the jaw was reported after oral surgical procedure or trauma [29]. Most of these complications have occurred in patients with cancer who have often received monthly high-dose BP infusions. To our knowledge, no data are available concerning the relationship between single-dose administration and the possible side effects of BPs.

In the present study, ZA was administered as a single dose of 0.1 mg/kg consistent with previous studies [14–16]. It has been proved that the plasma concentration of the drug gradually declines within 28 days [31]. Thus, a repeat dose of ZA could be administered 28 days after the initial single dose, if required. However, further studies are necessary to evaluate the effects of redosing on the physiological bone remodeling compared with the application of a single dose.

In conclusion, the result of the present experimental study has revealed that a temporary delay in physiological bone remodeling using single dose of ZA increases bone mineral content and makes the microarchitecture of the mandibular condyle more compact. These effects may be regarded as base data and considered in numerous clinical situations including TMJ.

## Figures and Tables

**Table 1:** Comparison of the densitometric data.

Group	Condyle (*n*)	Aluminum equivalent (mm) (mean ± SD)
Control	30	5.535 ± 2.754
ZA	30	9.676 ± 3.475

*P* = 0.001 (statistically significant).

**Table 2:** Comparison of the histomorphometric data.

Histomorphometric parameters	Control-condyle (*n* = 30)	ZA-condyle (*n* = 30)
Bone volume (%)	57.681 ± 15.95	75.483 ± 9.02*
Trabecular width (mcm)	54.188 ± 13.379	77.296 ± 18.352*
Trabecular thickness (mcm)	41.65 ± 11.547	58.017 ± 19.539*
Trabecular separation (mcm)	35.194 ± 12.025	16.721 ± 8.296*
Node-terminus ratio (NNd/NTm)	1.999 ± 1.399	3.159 ± 1.262*

*P < 0.05 (statistically significant).
